# Flavivirus Infection Associated with Cerebrovascular Events

**DOI:** 10.3390/v12060671

**Published:** 2020-06-22

**Authors:** Cássia F. Estofolete, Bruno H. G. A. Milhim, Nathalia Zini, Samuel N. Scamardi, Joana D’Arc Selvante, Nikos Vasilakis, Maurício L. Nogueira

**Affiliations:** 1 Department of Infectious, Dermatological and Parasitic Infections, Sao Jose do Rio Preto Medical School, Sao Jose do Rio Preto 15090-000, Brazil; cassiafestofolete@gmail.com (C.F.E.); brunohgam@hotmail.com (B.H.G.A.M.); nathalia_zini@hotmail.com (N.Z.); samuelns@gmail.com (S.N.S.); joanasselvante@gmail.com (J.D.S.); 2 Department of Pathology, University of Texas Medical Branch, 301 University Blvd., Galveston, TX 77555-0609, USA; nivasila@utmb.edu; 3 Center for Biodefense and Emerging Infectious Diseases, University of Texas Medical Branch, 301 University Blvd., Galveston, TX 77555-0609, USA; 4 Center for Tropical Diseases, University of Texas Medical Branch, 301 University Blvd., Galveston, TX 77555-0609, USA; 5 Institute for Human Infection and Immunity, University of Texas Medical Branch, 301 University Blvd., Galveston, TX 77555-0610, USA

**Keywords:** Zika virus, dengue virus, atypical manifestations, cerebrovascular events

## Abstract

Arthropod-borne viruses (arboviruses) of the genus *Flavivirus* are distributed globally and cause significant human disease and mortality annually. Flavivirus infections present a spectrum of clinical manifestations, ranging from asymptomatic to severe manifestations, including hemorrhage, encephalitis and death. Herein, we describe 3 case reports of cerebrovascular involvement in patients infected by dengue and Zika viruses in Sao Jose do Rio Preto, São Paulo State, Brazil, a hyperendemic area for arbovirus circulation, including dengue, yellow fever, chikungunya and Saint Louis encephalitis viruses. Our findings highlight the potential threat that unusual clinical manifestations may pose to arbovirus disease management and recovery.

## 1. Introduction

The genus *Flavivirus* includes several arboviruses that are considered significant threats to global public health [1], including dengue (DENV), Zika (ZIKV) and yellow fever (YFV) viruses. Over the past 70 years DENV spread throughout the tropics, reaching pandemic levels and putting at risk of infection over half of the world’s human population. By recent estimates, there are about 400 million dengue infections annually [2]. Conversely, ZIKV reached pandemic status within a span of a decade, starting with epidemics in the island of Yap, Federated States of Micronesia in 2007 [3] and its introduction and rapid spread in the New World as early as 2013 [4]. While most of flavivirus infections produce subclinical manifestations, clinical spectrum ranges from mild febrile illness to severe disease, characterized by hemorrhagic fever and neurologic involvement. Neurologic manifestations have been reported extensively in the literature [5,6,7,8,9,10,11,12,13,14,15,16] and were reviewed recently [17], described as congenital Zika syndrome (CZS) [18,19], Guillain-Barré syndrome (GBS) [7,8,10,20,21,22], encephalitis [23], transverse myelitis [24,25], encephalopathy [26,27,28], and at rare instances, cerebrovascular events [29,30,31,32,33,34,35].

Studies suggest that brain bleeding and strokes are associated to virus-induced and/or immune-mediated endothelium injury and platelet dysfunction [30,36,37]. The non-structural 1 protein (NS1), a well-conserved protein among flaviviruses, has been implicated to play an important role in vascular damage [38,39,40], suggesting that it may also play a role in the pathogenesis of cerebrovascular events. Although elucidating such a putative mechanism will be challenging, reporting cases based on complete clinical profiles, laboratory tests and cerebral images is a good start. Herein, we report 3 cases of flavivirus-associated acute neurological manifestations with cerebrovascular involvement, observed by our surveillance team in São José do Rio Preto (SJdRP), Brazil. The city of SJdRP is on the northwestern region of São Paulo State, has a tropical climate and is hyperendemic for various arboviruses, including DENV [25,41,42,43,44,45,46,47], ZIKV [43,48,49,50,51,52,53,54] and others [52,55,56].

## 2. Materials and Methods

### 2.1. Ethics Statement

These case series were submitted and approved by the Ethical Review Board (protocol number 28260620.2.0000.5415, 5 February 2020) of the School of Medicine of São José do Rio Preto (FAMERP), São Paulo, Brazil. Confidentiality was ensured by de-identifying of all questionnaires and samples before data entry and analysis.

### 2.2. Medical History and Sample Collection

Through an arbovirus surveillance system already stablished in the city, all dengue-suspected cases with warning signs (DwWS) or severe disease (SD) were monitored by our team from admission to discharge. Between November 2018 and June 2019, 31,534 cases were laboratory-confirmed as dengue in the city, of which 551 (551/31,534; 1.7%) cases were classified as DwWS or SD. Among them, 28 cases (28/31,534; 0.8%) were defined as SD according to the 2009 World Health Organization (WHO) dengue classification criteria [57], and 20 deaths were reported (20/31,534; 0.6%). Amongst these 28 severe cases, three presented cerebrovascular events and had clinical samples submitted for further diagnostic tests at the Laboratório de Pesquisas em Virologia (LPV), located within Medicine School of São José do Rio Preto (FAMERP), São Paulo State, Brazil. Demographic, epidemiological (gender, age) and clinical data (symptoms and radiologic observations) were obtained from electronic records. Blood and/or cerebrospinal fluid were collected, and results were reported the medical team.

### 2.3. Diagnostic Analyses

The samples were subjected to molecular and serological analyses, including Real Time Multiplex PCR (RT-PCR), Enzyme-Linked Immunosorbent Assay (ELISA) and Rapid Immunochromatographic assay (ICA), according to the sample collection time and onset of symptoms, following Brazilian and WHO guideline recommendations [57,58]. Details are provided below.

#### 2.3.1. Virus RNA Extraction and Real Time Multiplex PCR

Serum and CSF samples were used for viral RNA extraction and Real Time Multiplex PCR (RT-PCR). Briefly, virus RNA (vRNA) was extracted from 140 µL of sample using the Kit QIAmp^®^ Viral RNA (QIAGEN^®^, Germantown, MD, USA) following the manufacturer’s recommendations.

One-Step Real time multiplex PCR assays were performed using the GoTaq^®^ Kit by Promega. In fourplex reaction mixtures, 50 pmol (each) of DENV-1- and DENV-3-specific primers, 25 pmol each of DENV-2- and DENV-4-specific primers, and 9 pmol of each probe were combined in a 50-µL volume total reaction mixture. Real-time PCR was performed on a 96-well plate using the QuantStudio™ Dx instrument. Cycle threshold (Ct) values of less than 38 were interpreted as positive. Primer and probe sequences are available from the authors upon request [59].

#### 2.3.2. Enzyme-Linked Immunosorbent Assay (ELISA)

Serum samples were screened for exposure to dengue and Zika infection using the PanBio^®^ Dengue NS1 ELISA (Abbott, Santa Clara, CA, USA; former Alere Inc., Waltham, MA, USA)**,** human anti-DENV IgM ELISA (Abcam, Cambridge, UK) and the EUROIMMUN human IgM anti-ZIKV ELISA (EUROIMMUN, EURO-AG, Luebeck, Germany). CSF samples were screened using the NovaTech human anti-DENV IgM and anti-ZIKV IgM ELISA kits (NovaTech Immundiagnostica GmbH, Dietzenbach, Germany). All assays were performed according to the manufacturer’s recommendations using the corresponding positive and negative controls for quality control. ELISA plates were read using a Spectramax Plus ELISA reader at 450 nm (Molecular Devices, LLC, San Jose, CA, USA). Results were expressed in Standard Units (PU) and interpreted as <0.8 negative, >0.8 and <1.1 equivocal and >1.1 positive.

#### 2.3.3. Rapid Immunochromatographic Assay (ICA)

The SD BIOLINE Dengue DUO^®^ (Abbott, Santa Clara, CA, USA; former Alere Inc., Waltham, MA, USA) rapid immunochromatographic test kit was used for the detection of NS1 antigen and IgM and IgG DENV antibodies. Briefly, for NS1 antigen detection 100 µL of sample (serum or CSF) was applied to the corresponding well and allowed to incubate for 20 min. The presence or absence of antigen was determined by the absence or presence of the corresponding band. For the detection of IgM and IgG antibodies, 10 µL of sample (serum or CSF) was applied to the corresponding well, and 30 µL of diluent was added and allowed to incubate for 15–20 min. An NS1 antigen test was interpreted as positive by the presence of 2 color lines (T band and C band) within the 15–20 min incubation period regardless of which color line appears first and negative by the presence of 1 color line within the 15–20 min incubation period. An IgM or IgG antibody test is interpreted as positive when the control (C) line and the IgM (M) or IgG (G) line are visible and negative when only the control (C) line is visible.

## 3. Results

### 3.1. Case 1

**Figure 1:**
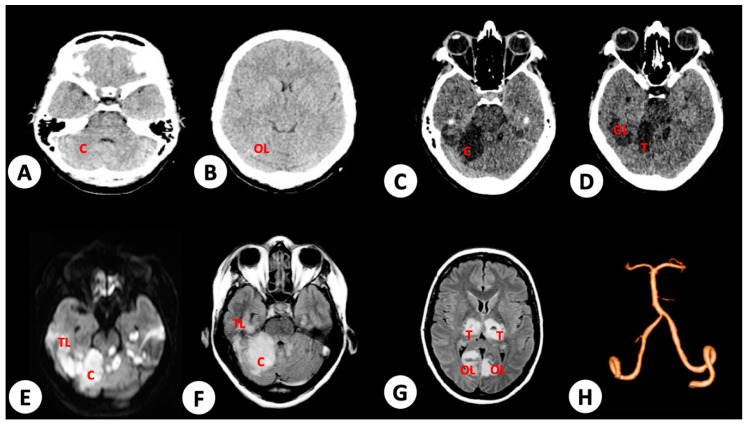
Brain radiologic imaging of patient 1. (**A**) No evidence of mass or vascular lesions in cerebellum or (**B**) occipital lobes (OL) in brain computed tomography (CT) immediately after seizure and decerebrate posturing events (10 days post onset of dengue symptoms). (**C**) T1 weighted image from brain nuclear magnetic resonance (NMR) performed 24 h after occurrence of disorientation and seizures, with evidence of ischemia in cerebellum (**C**) (11 days post onset of dengue symptoms). (**D**) Ischemic areas in thalamus (T) and occipital lobe. (**E**,**F**) Extensive ischemic infarct in cerebellum and parietal lobe (PL), in diffusion-weighted and fluid-attenuated inversion recovery (FLAIR) image from brain NMR performed 24 h after occurrence of acute clinical events (disorientation and seizures), respectively. (**G**) Ischemic infarct in thalamus and occipital lobe. (**H**) No evidence of vessel lesions, including basilar and vertebral arteries responsible to vascularization of injured areas in brain angiographic study performed 24 h after seizures.

A 38-year-old female without comorbidities was admitted complaining of fever, myalgia, arthralgia and ocular pain. Onset of symptoms were first observed 5 days prior to hospital admission. Upon initial examination by the medical team, she was conscious and with stable vital signs; however, she expressed abdominal pain and discomfort in the liver upon palpation. Her hematocrit was at 38% and platelet count at 16,000/mm³. On day 3 post admission, she tested positive to DENV by NS1 ELISA. Two days later, she suddenly became disoriented with seizures and decerebrate posturing. She was subjected to orotracheal intubation and admitted to the intensive care unit (ICU). A new serum sample obtained upon her admission to ICU was tested positive for DENV by IgM and IgG ELISA and ICA, respectively, and to DENV-2 by RT-PCR [59]. Her hematocrit declined to 33.4%, and the platelet count increased to 75,000/mm^3^. No acute mass or vascular lesions were observed by computed tomography (Figure 1A); however, multifocal ischemic infarctions were noted in the cerebellum, thalamus, and temporal and occipital lobes, associated to focal cerebral edema, in nuclear magnetic resonance (NMR) performed 24 h later (Figure 1B–H). Rheumatologic and coagulation diseases, as well as arrythmia and infectious endocarditis, were investigated as differential diagnoses and discarded. The patient gradually improved with supportive management care and was discharged after sixty days of hospitalization. Upon discharge, the patient retained limited motility (absence of voluntary movement of lower and upper extremities) and oral communication and required continuous home care support for basic daily duties.

### 3.2. Case 2

**Figure 2:**
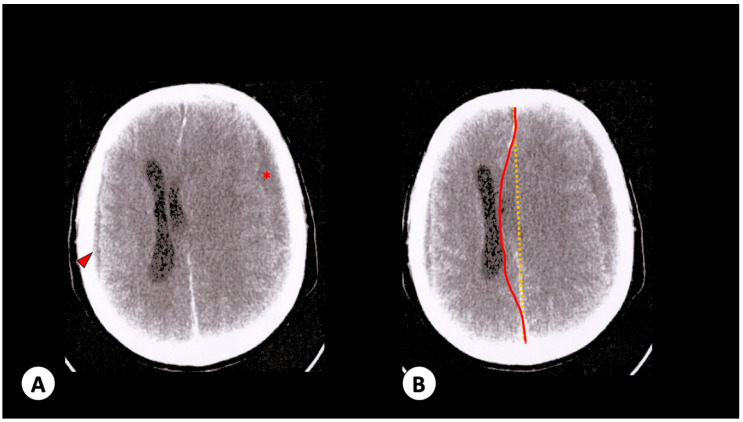
Patient 2 radiologic observations. (**A**) Brain no-contrast computed tomography with hypodense images in right parieto-occipital (0.5 cm—arrowhead) and left front-temporal (1.6 cm—red star) areas. (**B**) Corresponding brain contrasted computed tomography with middle-line deviation (red line) compared to regular condition (yellow line). It is not possible to view left lateral ventricular and cerebellar cisterns due to compression by left subarachnoid hemorrhage.

A 73-year-old male with diabetes and prostatic neoplasia with bone metastasis was admitted to our hospital with symptoms of myalgia, headache, nausea, vomiting, arthralgia and exanthema that appeared 2 days prior to his admission. He was conscious, communicating and with stable vital signs (hematocrit 24.9%, leukocytes 3500 cells/mm^3^ and platelets count 83,000/mm^3^). One day later, he developed mental confusion and decreased consciousness level. Immediate computed tomography showed evidence of hypodense images in right parieto-occipital (0.5 cm) and left front-temporal (1.6 cm) areas, middle-line deviation (1.5 cm) and left lateral ventricular and cerebellar cisterns compression, compatible as subarachnoid hemorrhage (Figure 2A,B). Hematocrit levels were of 22.4% and platelets count of 53,000 mm^3^ were recorded at the time. The patient was subjected to cerebral drainage, and his condition improved gradually. A serum sample tested positive by IgM DENV ELISA (Euroimmun AG, Lubeck, Germany). Seven days later, the patient exhibited sudden reduced mental awareness, cardiac arrest and death. The last laboratory exams showed hematocrit levels of 22% and platelet count of 35,000 mm^3^.

### 3.3. Case 3

**Figure 3:**
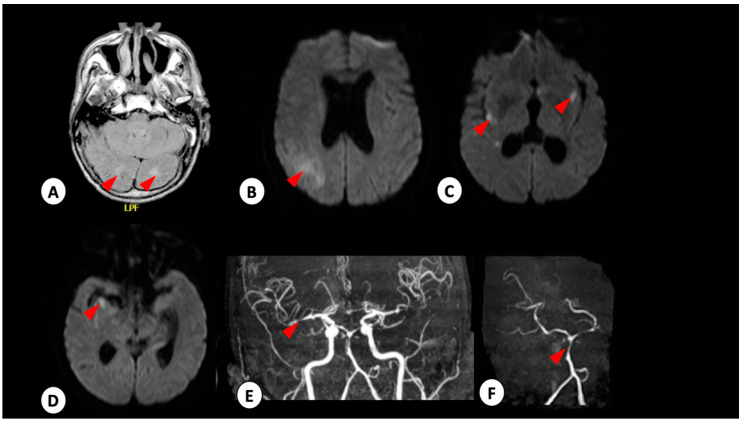
Patient 3 radiologic observations. Brain NMR performed 48 h after admission. Red arrowhead shows ischemic infarct in cerebellum (**A**), cerebellum (**B**), base nuclei (**C**) and hippocampus (**D**) in FLAIR (**A**) and diffusion-weighted image (**B**–**D**). (**E**,**F**) Stenosis in medium cerebral artery and stenosis in basilar artery, respectively.

A 65-year-old male presented to our hospital with symptoms of headache, altered consciousness level (confusion), drowsiness, dark urine and malaise that occurred a week prior to his admission. Upon admission, examination showed jaundice, disorientation, absence of neck rigidity or pupil alterations, a blood pressure of 158/90 mmHg and a heart rate of 78 bpm. Interview of his relatives indicated an absence of symptoms, including fever or dengue-like symptoms, up to the point of hospital admission and no recent travel history. NMR showed posterior meningium thickening (data not shown) and a reduced cerebral activity (mild, probably by the sedation) was observed in the electroencephalogram cerebral. A subsequent brain NMR performed 48 h later showed the presence of subarachnoid hemorrhage in the cerebellar tent, cerebellar sickle and right hippocampal groove (Figure 3A,B). Cerebral arteriography showed an aneurysm in A1–A2 angle of anterior cerebral artery (ACoA), right infusion and angiographic vasospasm, Fischer 1 Score (Figure 3C). Following the second resonance, the differential diagnosis was modified to subarachnoid hemorrhage with spontaneous posterior vessel constriction syndrome or vasculitis. Cerebrospinal fluid (CSF) was collected (leukocytes 83 cells/mm^3^ (79% lymphocytes), glucose 48 mg/dL, protein 43 mg/dL, bacteria culture negative). IgM capture ELISA on serum and CSF samples were both negative for DENV and positive for ZIKV. The patient was subjected to cerebral drainage, received supportive care treatment and 53-days-later was discharged, totally dependent on others for daily functions, such as hygiene care, food and mobility.

## 4. Discussion

In recent years, flavivirus-associated neurological manifestations have often been reported. However, their incidence rate remains unclear for both Zika and dengue [60,61], especially for cerebrovascular complications [60]. To date, our knowledge of cerebral hemorrhage and ischemic stroke in flavivirus infections has mainly been provided by case reports due to their low occurrence [17]. Herein, we report 3 cases of acute flavivirus infection-associated cerebrovascular events, two of them associated with dengue and one to Zika infections.

The current dengue clinical classification, proposed by WHO in 2009, includes central nervous system (CNS) involvement in the definition of SD requiring intensive care and supportive management [57]. DENV infections in endemic regions have long been recognized for their neuronal manifestations [62,63], where frequency ranges from 0.5% to 21% [60], and the incidence rate for strokes is less than 1% [30,37]. Generally, patients develop early non-specific symptoms such as fever, headache, myalgia and exanthema, with initial and transient resolution, followed by recrudescence of headache, fever and neurological symptoms onset [23], similar to our patient 1, who, in recovery phase, progressed to neuronal impairment.

Several factors may contribute to neurological manifestations of dengue, ranging from direct viral invasion of CNS to vascular and metabolic abnormalities [64], including prolonged shock, electrolyte disorders, cerebral edema or anoxia [65]. However, the pathogenesis of dengue neurological involvement remains unclear. Evidence supports that DENV may be neurovirulent [64,66,67,68] and invade the CNS [69,70], especially for infections attributed to DENV-2 and DENV-3 [62,64,71,72,73,74]. Furthermore, damage of the blood–brain barrier (BBB) during DENV infection in animal models corroborates the notion of viral invasion [75] into the brain prominently involving astrocytes, as well as microglia and endothelial cells (reviewed in [76]). Virus invasion of the brain may trigger T-cell activation and release of inflammatory mediators, as well as cytokine overproduction, such as interleukin (IL) 2, interferon (INF) γ and tumor necrosis factor (TNF) α, which could lead in immune-mediated endothelial damage [67,77,78,79,80]. Additionally, alterations in coagulation and viral-induced vasculitis might lead to cerebral hemorrhages and infarctions [80]. Thus, in cases of cerebrovascular events, meningovasculitis, a transient hypercoagulable state [31,33] or thrombocytopenia [31], elevated vascular permeability and plasma leakage [79,80] during dengue infection have been postulated as a possible mechanism of pathogenesis.

Similar to DENV, ZIKV neurovirulence and tropism have been well-established with a myriad of syndromes, such as CZS, GBS and others [81,82,83,84]. GBS has been implicated to both direct viral infection in neural tissues and autoimmune action against neurons and glia cells [85,86,87]. Furthermore, previous studies have demonstrated that endothelial cells of the BBB are permissive to ZIKV infection and replication, and it may penetrate the endothelium, causing a neuronal tissue damage [88,89].

The NS1 glycoprotein is involved in viral replication, immune evasion, and vascular leakage during flavivirus infection [90]. In addition to its membrane-bound form, NS1 may be secreted in patient serum during infection and widely used as a diagnostic tool for acute flavivirus infection [53,91]. Circulating levels of NS1 are correlated to DENV viremia and higher risk of SD [90]. The soluble and membrane-associated NS1 may activate complement system leading to generation of fragments such as anaphylatoxins that contribute to acute inflammation and, consequently, pathogenesis of vascular leakage that occur in severe manifestations of DENV infections [92]. In DENV-2-infected children, the greatest amount of plasmatic NS1 level was evidenced in those with more elevated viremia and free soluble NS1 level within 72 h from illness onset. Such findings were associated with risk of developing a more severe form of the disease [90]. This relationship between disease severity and NS1 protein has been corroborated in several studies [93,94,95].

A recent in vivo and in vitro study demonstrated that flavivirus secreted NS1 alters endothelial cell permeability and induces disruption of critical membrane components. While ZIKV NS1 has caused increased vascular permeability in both umbilical vein and developing brain endothelial cells, DENV NS1 has induced hyperpermeability in more tissues, such as the lung, skin, liver, brain and placenta [40]. Thus, NS1-induced hyperpermeability plays an important role in the development of SD and appears to be tissue-associated, suggesting a differential ability in leading to endothelial dysfunction [38,40]. A proposed mechanism in a mouse model of infection suggests that DENV NS1 elicits an inflammatory cytokine response (e.g., TNF-a, IL-6, IFN-*β*, IL-1*β* and IL-12) and endothelial cell monolayer permeability via toll-like receptor (TLR) 4 activation [39] and effect is attached by glycosylation-dependent DENV NS1 endocytosis, similar to ZIKV and West Nile virus (WNV) [96]. Furthermore, the application of the TLR4 antagonist *Rhodobacter sphaeroides* lipopolysaccharide (LPS-RS) inhibits vascular leakage [39]. Since NS1 plays a direct role in endothelial dysfunction and disruption leading to SD, it may also play a role in the development of cerebrovascular events in flavivirus infections. In our first case, the onset of neurological symptoms onset occurred on day 7, a critical phase of in dengue course, although NS1 level peaked in the serum on day 5, suggesting a delayed effect on the cerebrovascular endothelium.

While the clinical association between flavivirus and cerebrovascular events is challenging, obtaining a differential diagnosis is even more challenging. According to national and international guidelines, the presence of NS1 antigen, DENV RNA and/or anti-dengue IgM is enough for diagnosis of acute infection [57,58]. As presented in case 1, by the time stroke was diagnosed on our patient, we confirmed acute dengue infection by serology (positive NS1 and IgM dengue ELISA). Similarly, patient 2 was positive for acute DENV infection (positive IgM dengue ELISA), presented with similar clinical manifestations that progressed to a stroke. In a recent surveillance study in Brazil, Vieira et al. [97] suggested a link of DENV and ZIKV infection to development of neurologic syndromes, as encephalitis, acute transverse myelitis, and GBS. Although NS1-induced permeability may be involved to the development of neurological syndromes, the sensitivity of ELISA NS1 tests is influenced by the timing of testing after onset of symptoms, as well as the serological status of patients [98,99]. Thus, the diagnosis of a vascular event, during dengue acute infection, without coagulation or autoimmune disorders, led us to conclude that DENV infection had been associated with cerebrovascular events (multifocal ischemic infarctions and focal cerebral edema in patient 1 and stroke in patient 2).

Cerebrovascular events in ZIKV-infected patients are even less frequent than in dengue infected patients [35]. Considering that Zika has tropism and may infect endothelial cells, as demonstrated in models using human vein and artery cells [89,100,101], and due to its phylogenetic relationship to DENV in genus *Flavivirus* [102], and the eventual similarity among their components, including NS1 [103], it is reasonable to admit a correlation between this protein and strokes or brain hemorrhage. Besides, ZIKV-infected monocytes may have transmigrated through BBB in a model based on inflammatory activation, with essential role of T and B lymphocytes, TLR, receptor for advanced glycation end products (RAGE) and microvascular endothelial dysregulation [104]. The end of RAGE pathway is the production of IL-1*β* among other cytokine by monocytes [105]. The IL-1*β* upregulation was observed in human brain microvascular endothelial cells after ZIKV infection, followed by decreased proinflammatory cytokine levels when IL-1 receptor antagonist was added to model [106]. Once the integrity of BBB, which is composed of specialized microvascular endothelial cells, is crossed, the virus is able to reach and infect its target cells, and thus, presentation of rare clinical events may be observed [107]. Indeed, a number of studies have shown the prominent role of astrocytes in ZIKV infection [108,109,110,111,112], which may play a role in ZIKV infection in the brain.

Because of ZIKV short viremia, serum RNA-negative results do not exclude recent ZIKV infection. For this reason, since February 2016, when Zika emergence became a public health concern [113], the Centers for Disease Control and Prevention’s (CDC) Zika Immunoglobulin M antibody capture enzyme-linked immunosorbent assay (Zika MAC-ELISA) has been significantly improved and used for the presumptive diagnosis in human serum or CSF. However, patients with recent flavivirus natural infection, such as DENV or vaccinated against yellow fever or Japanese encephalitis may test positive [114]. In a recent study, 3 out of 74 patients with neurological syndromes have been Zika-diagnosed, based on anti-Zika IgM in CSF [97]. According to the 2015 CDC guidelines, specific arbovirus neuroinvasion is confirmed by demonstrating both a virus-specific antibody in CSF, as well as a negative result for other arbovirus-specific antibodies [115]. Both requirements were met in case 3, in which we detected the presence of ZIKV-specific antibodies in CSF and absence of DENV-specific antibodies.

Both DENV and ZIKV may infect the central nervous system through a hematogenous route by crossing the BBB facilitated by the inflammatory response and permeability increase [116]. Although the exact pathway through which a flavivirus invades the CNS remains unclear, the complex interaction between virus and host immune response serves as the basis to neuropathogenesis, as demonstrated in other flavivirus infections, such as Japanese encephalitis virus (JEV) and WNV [76]. Identifying such cases is a crucial tool to understand the broad clinical spectrum of common and unusual arboviruses and their public health impact.
